# More DNA and RNA of HBV SP1 splice variants are detected in genotypes B and C at low viral replication

**DOI:** 10.1038/s41598-021-03304-w

**Published:** 2021-12-13

**Authors:** Ka-Cheung Luk, Jeffrey Gersch, Barbara J. Harris, Vera Holzmayer, Dora Mbanya, Silvia Sauleda, Mary A. Rodgers, Gavin Cloherty

**Affiliations:** 1 Dept. 9NC, Infectious Diseases Research, Abbott Laboratories, Bldg. AP20, Rm 301E, 100 Abbott Park Road, Abbott Park, IL 60064 USA; 2 University of Yaounde I, Yaounde, Cameroon; 3 University of Bamenda, Bamenda, Cameroon; 4 Banc de Sang I Teixits, Barcelona, Spain

**Keywords:** Cancer, Molecular biology, Biomarkers, Gastroenterology, Medical research

## Abstract

HBV produces unspliced and spliced RNAs during replication. Encapsidated spliced RNA is converted into DNA generating defective virions that are detected in plasma and associated with HCC development. Herein we describe a quantitative real-time PCR detection of splice variant SP1 DNA/RNA in HBV plasma. Three PCR primers/probe sets were designed detecting the SP1 variants, unspliced core, or X gene. Plasmids carrying the three regions were constructed for the nine HBV genotypes to evaluate the three sets, which were also tested on DNA/RNA extracted from 193 HBV plasma with unknown HCC status. The assay had an LOD of 80 copies/ml and was equally efficient for detecting all nine genotypes and three targets. In testing 84 specimens for both SP1 DNA (77.4%) and RNA (82.1%), higher viral loads resulted in increased SP1 levels. Most samples yielded < 1% of SP1 DNA, while the average SP1 RNA was 3.29%. At viral load of ≤ 5 log copies/ml, the detectable SP1 DNA varied by genotype, with 70% for B, 33.3% for C, 10.5% for E, 4% for D and 0% for A, suggesting higher levels of splicing in B and C during low replication. At > 5 log, all samples regardless of genotype had detectable SP1 DNA.

## Introduction

Hepatitis B virus (HBV), which can cause chronic liver disease that may result in cirrhosis and hepatocellular carcinoma (HCC), is a hepadnavirus with a 3.2 kb, relaxed circular, partially double-stranded DNA genome which is converted into covalently closed circular DNA in the nucleus of infected hepatocytes. Transcription on the double-stranded HBV DNA genome by host RNA polymerase generates a 3.5 kb precore/pregenomic RNA (pgRNA) and three subgenomic RNAs (2.4, 2.1 and 0.7 kb). The pgRNA serves as an encapsidated template for reverse transcription into DNA during genome replication and as mRNA for the production of core and polymerase (Pol) proteins. The precore mRNA is slightly longer and encodes hepatitis B e antigen (HBeAg). Subgenomic 2.4 kb and 2.1 kb mRNAs encode the HBV surface proteins (HBs), and the small 0.7 kb mRNA encodes the X protein^1^.

In addition to these unspliced wild-type (wtHBV) RNAs, a series of at least 18 spliced (SP) HBV (spHBV) RNAs derived from the 3.5 kb pgRNA have been identified in sera and livers of patients with chronic hepatitis B (CHB)^2–4^. The most frequently detected spHBV variant is a 2.2 kb molecule termed as SP1. The SP1 splice variant is generated through the excision of a 1.2 kb intron from the pgRNA with 5’ donor and 3’ acceptor sites positioned at 2447/2448 nt and 488/489 nt, respectively. The SP1 species was found to account for up to 30% of total 3.5 kb pgRNA in HBV-infected hepatoma cell lines and for 50–60% of all splice variants detected in HBV patient samples^2,5–7^. Similar to the unspliced pgRNA, HBV spliced RNA variants can also be encapsidated and reverse transcribed by the HBV viral polymerase into DNA to produce defective HBV (dHBV) viral particles, with replication and envelopment requiring polymerase and envelope proteins supplied *in trans* by wild-type HBV^5,8,9^. The level of dHBV particles in the sera of CHB patients has been related with liver disease^10,11^ and was increased prior to the development of HCC^12^. This singly spliced SP1 RNA encodes a novel protein known as hepatitis B spliced protein (HBSP)^13,14^, which is a fusion product of the first 46 amino acid residues of the viral polymerase and 47 amino acid residues from a new open reading frame (ORF) created by the splicing event. HBSP has been linked to an increased risk of the development of HCC by promoting viral replication, protein production and liver fibrosis^14–16^. In addition to the singly spliced SP1 variant, three doubly spliced HBV variants, SP2, SP4, and SP15, possess the same SP1 1.2 kb excision, as well as a second smaller excision in the core gene^2,4,12^.

The clinical course and long-term outcome of HBV infection are influenced by several factors including viral genotype, DNA viral load over time, and specific viral mutations^17^. Nine genotypes (A to I) of HBV worldwide have been identified based on divergence over the entire genomic sequence of more than 8%^18,19^. HBV genotypes have specific geographic distributions^20,21^: genotypes A and D are prevalent in Africa, India and Europe; B and C are widely dominant in Asia–Pacific; E is widely found in West Africa; F is mainly seen in South and Central Americas; G is prevalent in the United States and France; H is found in Central America; and I is largely restricted to Vietnam and Laos. Many studies have been published regarding the relationship between HBV genotype and serious sequelae of HBV infection, including cirrhosis and HCC. Infection with genotype B has been found to be associated with the development of HCC and relapse of HCC in younger age, whereas infection with genotype C has been frequently linked to an increased risk of HCC in the older age (> 40 years)^22^. Genotypes B and C cause more severe cellular damage than A and D^23,24^ and are more oncogenic^20,25–28^. While the mechanism of pathogenicity of different HBV genotypes is still not fully understood, it has been reported^6,29^ that different HBV genotypes produce different levels of HBV splice variants, which are predictive of liver cancer^12,30^.

HBV splicing is a common event during chronic infection which has been shown to be associated with the development and recurrence of HCC^12,30^ and impairing patient response to interferon treatment^4^, therefore measurement of the HBV splicing level may serve as a valuable tool in identifying CHB patients who are at increased risk of developing HCC. Herein we describe the development of an automated, high-throughput, sensitive, quantitative, real-time PCR assay on the Abbott *m*2000*sp*/*rt* system for detecting DNA and RNA of the most common spliced variant SP1 and associated variants (SP2, SP4, and SP15) in plasma of CHB patients. In addition, we observed that HBV genotypes B and C generated more detectable splice variants than A, D or E during low replication (≤ 5 log copies/ml).

## Results

### Quantification of unspliced core (wild-type), SP1 spliced and X gene (total) HBV DNA by real-time PCR

**Figure 1:**
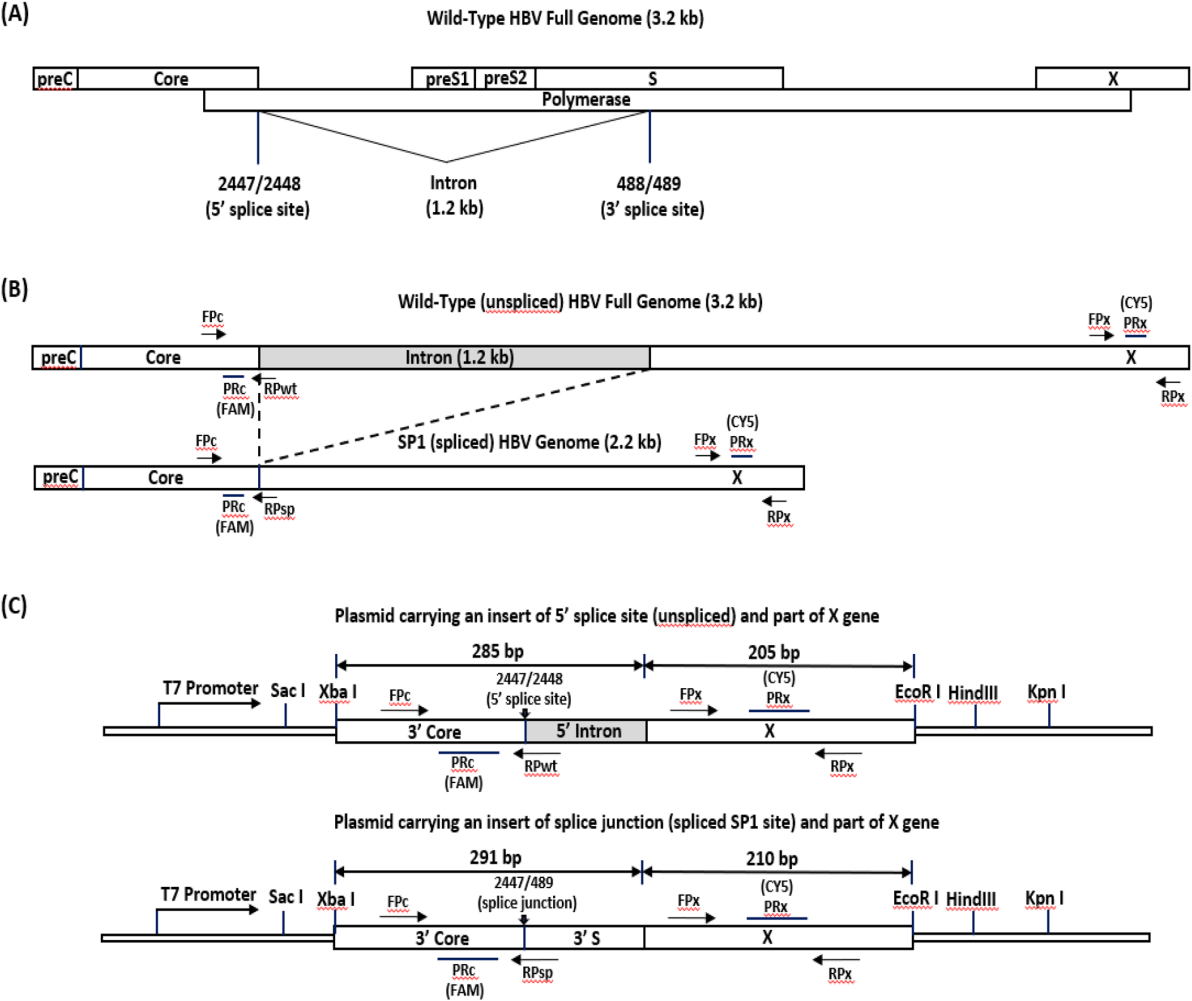
HBV genome and real-time PCR strategy. (**A**) HBV wild-type full genome with SP1 5’ splice site, 1.2 kb intron and 3’ splice site. (**B**) HBV genomes before (3.2 kb) and after (2.2 kb) the SP1 splicing. (**C**) Plasmid constructs carrying an insert (XbaI—EcoRI) of either HBV 5’ splice site (unspliced) or SP1 splice junction and the 5’ end of the X gene. The locations of real-time PCR primers and probes to detect the 5’ splice site, SP1 splice junction and X gene are shown. Using B-M54923 as an HBV reference sequence, the core gene forward primer (FPc) is located at nt 2365—2384, the core gene reverse FAM probe (PRc) is at nt 2428—2402, the core gene reverse primer (RPwt) designed to cross the 5’ splice site to specifically detect the unspliced wild-type HBV full genome is at nt 2465—2444, the shifted reverse primer (RPsp) designed to cross the SP1 splice junction region to specifically detect the spliced SP1 HBV genome is at nt 507—489 ^ 2447—2444, the X gene forward primer (FPx) is at nt 1528—1547, the X gene forward CY5 probe (PRx) is at nt 1561—1581, and the X gene reverse primer (RPx) is at nt 1600—1582.

To establish an in vitro system to optimize real-time PCR conditions to quantitatively detect the HBV unspliced core/wild-type, SP1 spliced, and X gene/total DNA, we designed two sets of plasmid constructs (Fig. 1C): one set carrying an insert of 5’ splice site (unspliced/wild-type) located at the 3’ end of the core gene (Fig. 1A) and the 5’ end of the X gene, and another set having an insert of splice junction (spliced SP1 site) (Fig. 1B) and the 5’ end of the X gene. Three sets of real-time PCR primers and probes were designed to target the conserved regions within the 3’ end of the core gene and the 5’ splice site (the reverse primer RPwt crossing the 5’ splice site), the 3’ end of the core gene and the SP1 splice junction (the reverse primer RPsp crossing the SP1 splice junction), and the 5’ end of the X gene, respectively (Fig. 1B,C). Representative sequences from all nine HBV genotypes (A–I) were used to make the two sets of plasmid constructs to evaluate the performance of these three primers/probe sets. Each target plasmid was tested at 10^1^, 10^2^, 10^3^, 10^4^, 10^5^, and 10^6^ copies per PCR reaction. As shown in Fig. S1, all three regions from each of the nine genotypes were detected down to 10 copies/reaction. Similar threshold cycles (Cts) were observed across all nine genotypes and three regions, demonstrating that these three primers/probe sets detected each of them equally well. As an example, Fig. S2 shows the actual data of six standard curves derived from the amplification curves of tenfold serial dilutions (10^1^–10^6^ copies/reaction) of plasmids carrying the X gene, unspliced/wild-type core gene, or SP1 spliced core of genotypes A or C. Expectedly, Ct values for each copy number were consistent across the six standard curves. The six values of correlation (R^2^) of greater than 0.9970 demonstrated good linearity across the tested range.

Critically, the set of primers/probe for the detection of SP1 spliced plasmid DNA was unable to detect unspliced wild-type core plasmid DNA, and vice versa, thus demonstrating the specificity of these two sets of primers/probe (Fig. S3). The design of primers/probe set for the detection of SP1 spliced plasmid DNA was able to detect the singly spliced variant SP1 from the HBV plasma samples, but should also theoretically be able to detect doubly spliced variants SP2, SP4 and SP15, which have the same SP1 1.2 kb excision.

The sensitivity of the present assay was evaluated on a clinical plasma sample CHU2247 (sample #10 in Table S2) having a large volume and high titer of HBV X gene (8.49 log copies/ml) identified in this study. The limit of detection (LOD) was established by testing percentage of detection of three sets of lowest dilutions (40, 80 and 160 copies/ml). As elucidated in Table S1, 80 copies/ml was the lowest dilution at which > 95% of replicates were detected and thus accepted as the LOD for this assay.

### Evaluation of assay performance by testing 84 HBV DNA positive plasma samples for the presence of the X gene, unspliced core, and SP1 spliced HBV DNA

**Figure 2:**
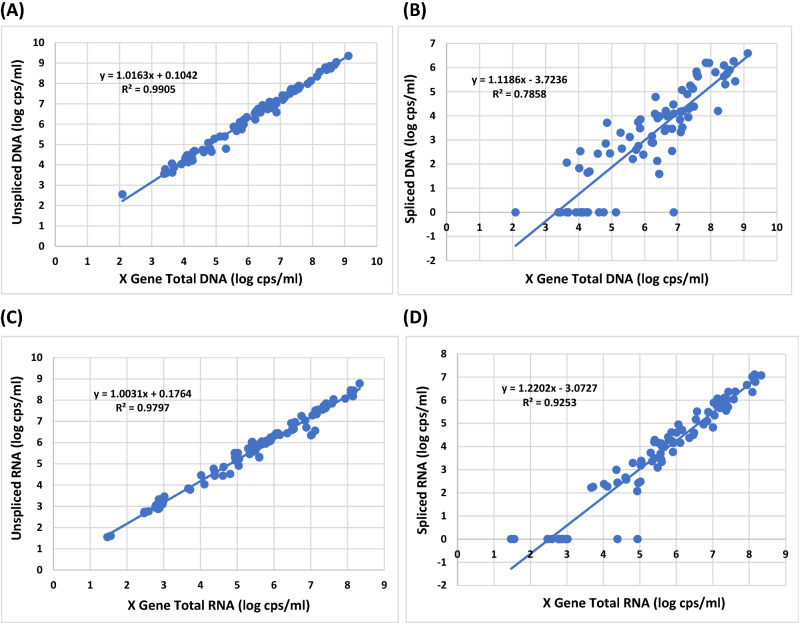
Relationship between the viral titer of HBV X gene and that of unspliced or spliced core gene based on the data of the 84 plasma specimens in Table S2. (**A**) X gene total DNA vs unspliced core DNA. (**B**) X gene total DNA vs SP1 spliced core DNA. (**C**) X gene total RNA vs unspliced core RNA. (**D**) X gene total RNA vs SP1 spliced core RNA.

**Figure 3:**
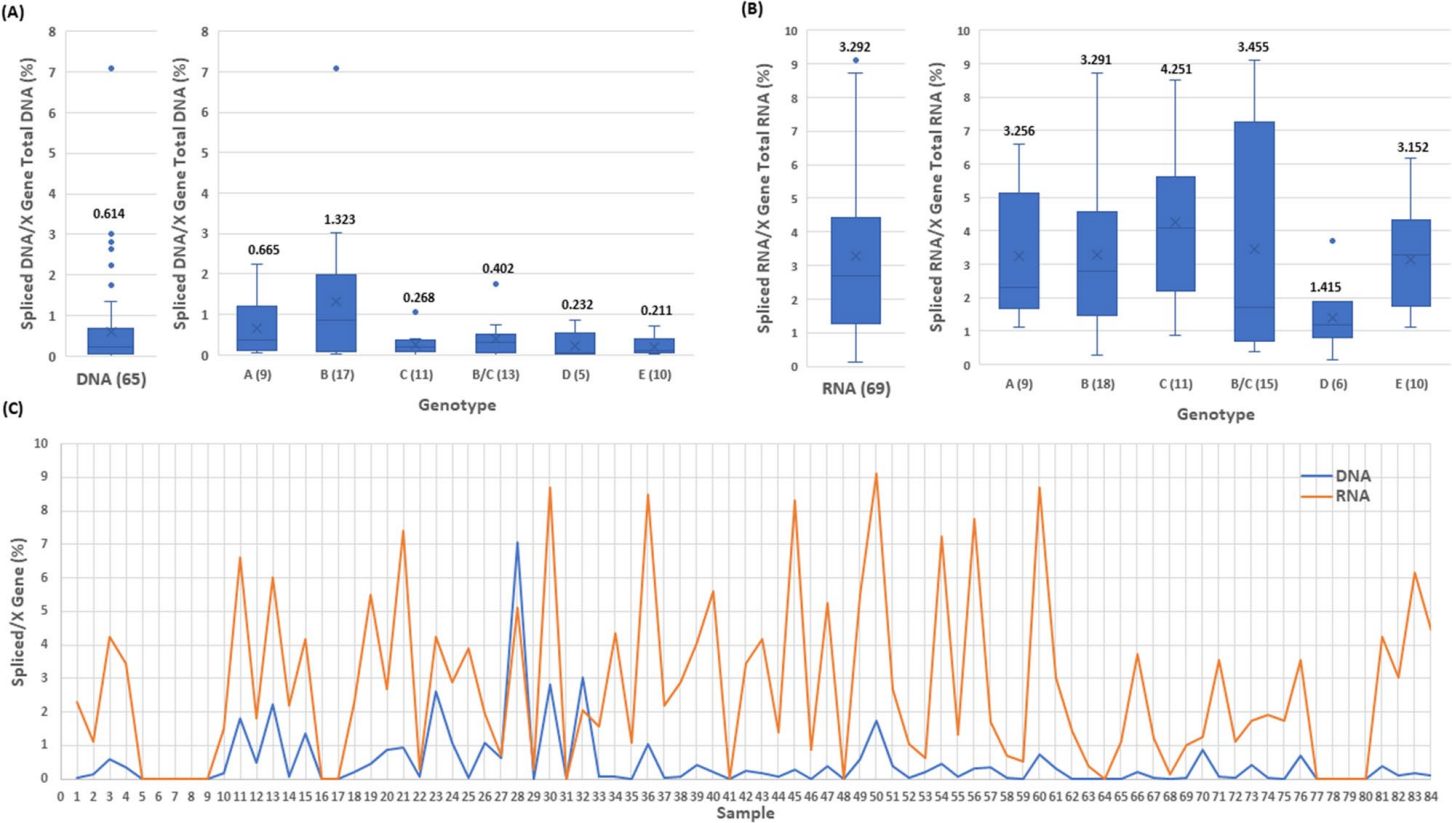
Distribution of the percentages of spliced DNA/X gene total DNA or spliced RNA/X gene total RNA based on the data of Table S2. (**A**) Comparison of the average percentages of spliced DNA/X gene total DNA. (**B**) Comparison of the average percentages of spliced RNA/X gene total RNA. (**C**) Comparison of DNA with RNA for each of the 84 samples for the percentages of spliced/X gene.

The utility of the developed molecular assay to detect the X gene (total HBV DNA), unspliced core (wild-type/wtHBV), and SP1 spliced (SP1, SP2, SP4 and SP15 spHBV) DNA was evaluated by testing 84 diverse HBV DNA positive clinical plasma specimens (including genotypes A, B, C, B/C recombinants, D and E) collected in Cameroon, France, Germany, Italy, Spain, Thailand, and Vietnam. These plasma samples were obtained from blood donors whose HCC status was not known. As tabulated in Table S2, the X gene DNA was detected in all 84 samples with the titers (viral loads) ranging from 2.09 to 9.12 log copies/ml, while the wild-type unspliced core DNA was also detected in all 84 samples with a similar range of 2.55 to 9.35 log copies/ml. The plot of unspliced core DNA viral load vs X gene total DNA viral load demonstrates a strong linear relationship with a high correlation value (R^2^) of 0.9905 and slope of 1.02 (Fig. 2A), reflecting that an extremely low level of spliced DNA, if any, was in circulation among these 84 samples. As for the titers of SP1 spliced DNA displayed in Table S2, a total of 65 of 84 (77.4%) samples were found to have detectable spliced DNA ranging between 1.59 to 6.59 log copies/ml, which was significantly lower than the titer range found in unspliced core DNA. When the 84 titers of the X gene DNA were plotted against those of the spliced DNA, a less linear relationship with an R^2^ value of only 0.7858 and a slope of 1.12 was observed (Fig. 2B), most likely driven by a high number of samples having no splicing at low viral loads. However, at high viral loads the level of HBV DNA splicing corresponded to the increased level of viral load. In fact, at viral loads of higher than 5 log copies/ml, all samples with an exception of two were found to have detectable spliced DNA. Conversely, at low viral loads of ≤ 5 log copies/ml of HBV DNA, 17 of 26 (65.4%) samples had no detectable spliced DNA. When the ratio of spliced DNA to X gene DNA was calculated for each of the 65 spliced DNA positive samples, the range of 0.001–7.079% was observed (Table S2) with 54 of 65 (83.1%) samples having less than 1% spliced DNA. The average level of spliced DNA was 0.614% among the 65 spliced DNA positive samples (Fig. 3A), demonstrating that SP1 spliced DNA variants represent a very minor population in the plasma of these blood donors in comparison with the unspliced wild-type HBV DNA genomes.

### Evaluation of assay performance by testing the same panel of 84 HBV DNA positive plasma samples for the presence of the X gene, unspliced core, and SP1 spliced HBV RNA

In addition to DNA detection, the usefulness of this molecular assay to detect the X gene, unspliced core, and SP1 spliced HBV RNA was also evaluated by testing the same panel of 84 HBV plasma specimens. As listed in Table S2, the X gene RNA (total) similar to the results found at the DNA level was also detected in all 84 samples with the titers ranging from 1.47 to 8.33 log copies/ml, and the wild-type unspliced core RNA was also detected in all 84 samples with a similar range of 1.55 to 8.78 log copies/ml. Similar to the DNA results, a strong linear relationship with an R^2^ value of 0.9797 and a slope of 1 was also observed between the X gene and unspliced RNA viral titers (Fig. 2C), thus implying that the level of spliced RNA was also very low. As for the titers of SP1 spliced RNA shown in Table S2, a total of 69 of 84 (82.1%) samples had detectable spliced RNA ranging between 2.07 to 7.11 log copies/ml. When the 84 titers of the X gene RNA were compared to those of the spliced RNA, a linear relationship with an R^2^ value of 0.9253 was observed (Fig. 2D), higher than seen in the plot at the DNA level (Fig. 2B). This RNA comparison demonstrated that HBV splicing was related to HBV replication. At viral loads above 5 log copies/ml, all samples were found to have detectable spliced RNA (Fig. 2D). On the other hand, with the titers of ≤ 5 log copies/ml of HBV RNA, 15 of 26 (57.7%) samples generated no detectable spliced RNA. These results were consistent with those observed for the spliced DNA (Fig. 2B). When the ratio of spliced/X gene RNA was calculated for each of the 69 spliced RNA positive samples, the range of 0.138–9.12% was observed (Table S2) with only 9 of 69 (13%) samples having less than 1% SP1 spliced RNA. The average level of spliced RNA was 3.292% among the 69 spliced RNA positive samples (Fig. 3B), more than 5 times higher than that of spliced DNA (Fig. 3A). Nevertheless, the level of spliced RNA variants remained very low in this panel of HBV specimens in comparison with the unspliced wild-type HBV RNA genomes.

As illustrated in Fig. 3C, except for two samples (#28 and #32), the spliced/X gene RNA ratio was significantly higher than the spliced/X gene DNA ratio in each of the remaining 63 spliced DNA positive samples. Four samples among the 84 samples in this panel tested negative for spliced DNA but positive for spliced RNA with low titers ranging from 2.27 to 3.33 log copies/ml, suggesting that spliced RNA due to its relatively higher level potentially is a stronger biomarker for monitoring HBV splicing than spliced DNA.

### Effect of HBV genotypes on HBV SP1 DNA/RNA splicing

The diversity of genotypes in the panel of 84 HBV specimens, including genotypes A, B, C, B/C recombinants, D, and E, allowed for investigation into the relationship between HBV genotypes and HBV SP1 splicing. Figure 3A shows a box plot of spliced/X gene DNA percentages sorted according to the six HBV genotypes. All except for genotype B had an average level of less than 1%. The average level of spliced DNA among the 17 genotype B samples was 1.323%, with one sample generating 3.02% and another one yielding 7.079%, suggesting that genotype B had a relatively higher level of HBV DNA splicing than A, C, B/C, D, or E. Figure 3B presents the genotype-specific breakdown of spliced/X gene RNA ratios. Except for D with an average level of 1.415%, all other five genotypes showed similar average percentages (3.152–4.251%). However, genotypes B (7.413 and 8.71%), C (8.318 and 8.511%), and B/C (8.71 and 9.12%) included individual samples generating higher percentages of spliced RNA than A (6.026 and 6.607%), D (3.715%) and E (4.467 and 6.166%) (Table S2), thus showing that genotypes B, C, and B/C had relatively higher levels of HBV RNA splicing than A, D or E in individual samples to produce higher percentages of spliced RNA variants.

**Figure 4:**
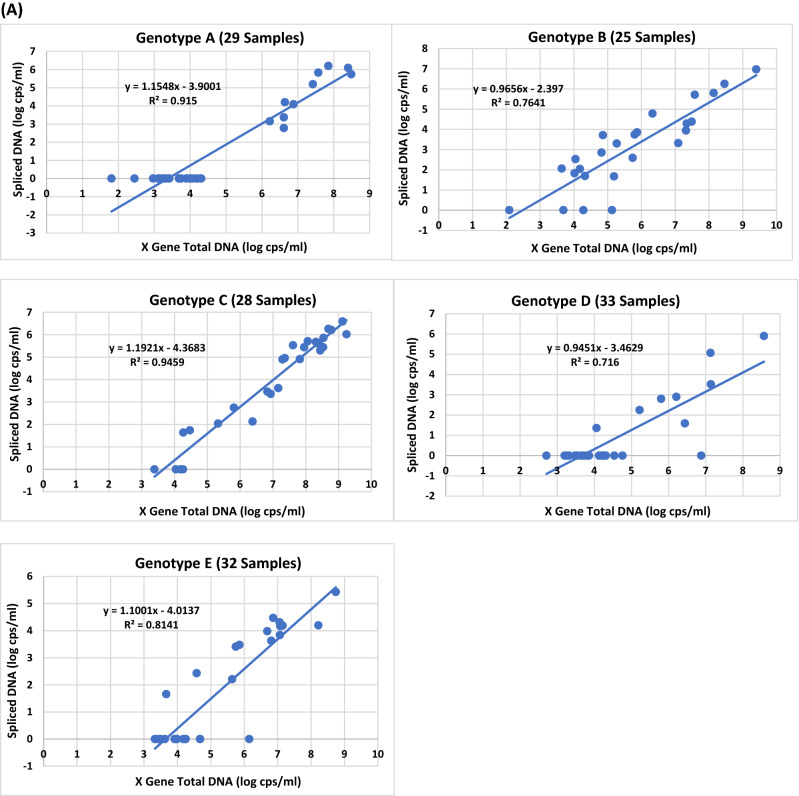

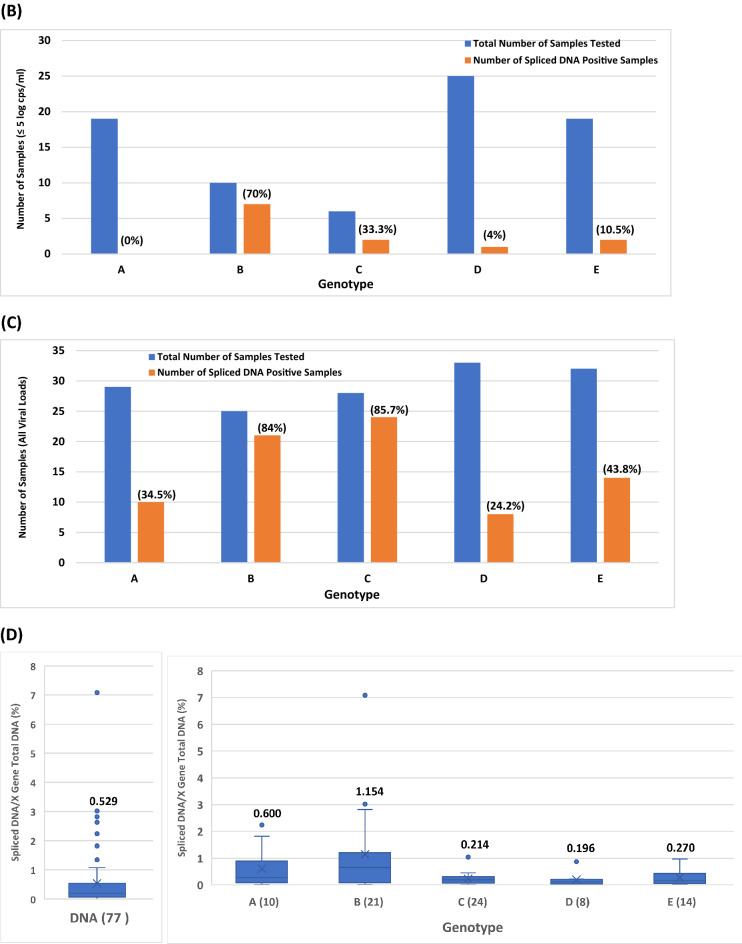
The effect of HBV genotypes on the viral titers of SP1 spliced DNA based on the data of the 147 plasma samples in Table S3. (**A**) Relationship between the viral titer of X gene total DNA and that of SP1 spliced DNA for genotypes A–E. (**B**) Comparison of the numbers of spliced DNA positive samples detected from 79 of the 147 samples (A–E) with a viral load of ≤ 5 log copies/ml. The percentage of detectable spliced DNA positive samples is denoted in parentheses for each genotype. (**C**) Comparison of the numbers of spliced DNA positive samples detected from all of the 147 samples (A–E) regardless of viral load. The percentage of detectable spliced DNA positive samples is denoted in parentheses for each genotype. (**D**) Comparison of the average percentages of spliced DNA/X gene total DNA.

To obtain more conclusive data on the impact of genotypes on HBV splicing, 78 additional HBV plasma samples from blood donors with unknown HCC status were analyzed for the presence of SP1 spliced DNA. Table S3 shows the viral titers of X gene DNA and SP1 DNA from 147 samples (A, B, C, D and E) comprising 69 (in bold) of set 1 from Table S2 and 78 (in italics) additional specimens. The plots of X gene vs spliced DNA titers were shown in Fig. 4A for genotypes A (29 samples), B (25), C (28), D (33), and E (32). Again, all five plots confirmed previous observations that higher viral titers increased the levels of HBV DNA splicing. At high viral loads (> 5 log copies/ml), almost all samples were found having detectable spliced HBV DNA, regardless of genotype. By enumerating the spliced DNA positive samples at low viral loads (≤ 5 log copies/ml) for the five genotypes, A (0/19 = 0%), D (1/25 = 4%) and E (2/19 = 10.5%) produced a low percentage of detectable SP1 DNA positive samples, while B (7/10 = 70%) and C (2/6 = 33.3%) conversely generated a higher percentage of detectable spliced DNA positive samples (Fig. 4B), indicating that genotype B along with C in this 147-sample panel yielded a significantly higher percentage of detectable spliced DNA positive samples than A, D or E during low replication (≤ 5 log copies/ml). These observations remained consistent when all samples irrespective of viral load were included; the percentages of SP1 DNA variants detected in B (84%) and C (85.7%) were again significantly higher than those in A (34.5%), D (24.2%), or E (43.8%) (Fig. 4C). As for spliced/X gene DNA ratios, the average for the 77 spliced DNA positive samples found in this 147 sample-panel was 0.529% (Fig. 4D). Among the five genotypes, B (21 samples) expectedly not only had the highest average percentage (1.154%), but also the individual samples with the two highest percentages (3.02 and 7.079%) of SP1 DNA.

**Figure 5:**
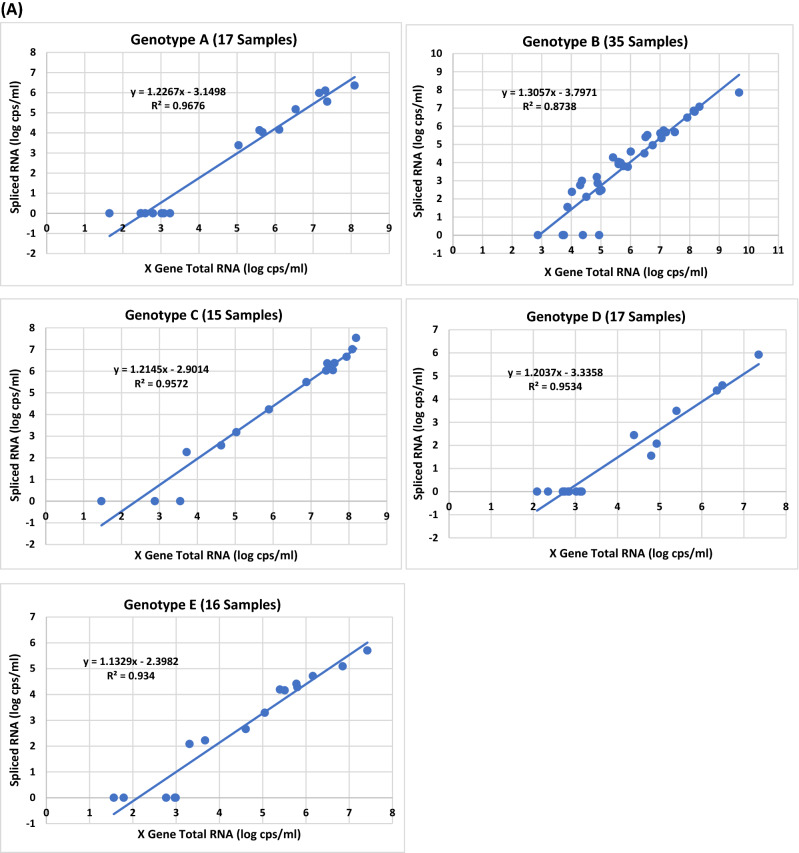

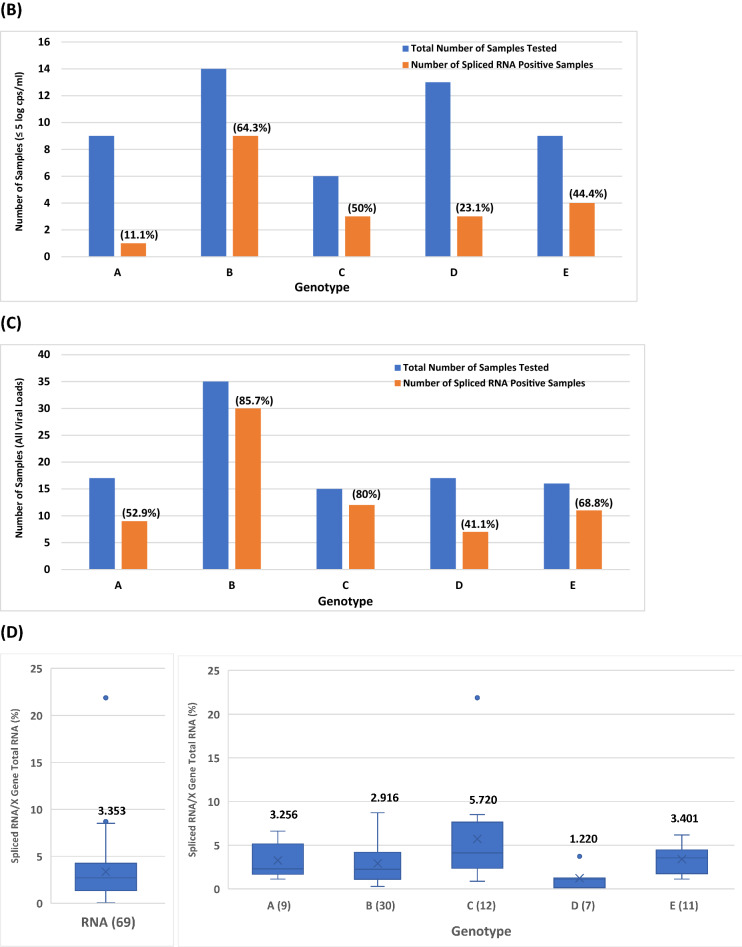
The effect of HBV genotypes on the viral titers of SP1 spliced RNA based on the data of the 100 plasma samples in Table S4. (**A**) Relationship between the viral titer of X gene total RNA and that of SP1 spliced RNA for genotypes A–E. (**B**) Comparison of the numbers of spliced RNA positive samples detected from 51 of the 100 samples (A–E) with a viral load of ≤ 5 log copies/ml. The percentage of detectable spliced RNA positive samples is denoted in parentheses for each genotype. (**C**) Comparison of the numbers of spliced RNA positive samples detected from all of the 100 samples (A–E) regardless of viral load. The percentage of detectable spliced RNA positive samples is denoted in parentheses for each genotype. (**D**) Comparison of the average percentages of spliced RNA/X gene total RNA.

As with SP1 DNA splicing, 31 additional HBV plasma samples different from the 78 samples analyzed in Table S3 were tested for the presence of SP1 RNA variants. Table S4 shows the viral titers of X gene RNA and SP1 RNA from 100 samples (A, B, C, D and E) comprising 69 (in bold) of set 1 from Table S2 and 31 (in italics) additional specimens. The titers of X gene vs spliced RNA were compared (Fig. 5A) for genotypes A (17 samples), B (35), C (15), D (17), and E (16). Again, at high viral loads of > 5 log copies/ml, all samples were found to have detectable SP1 RNA irrespective of genotype. As for the spliced RNA positive samples at low viral loads (≤ 5 log copies/ml) for the five genotypes, A (1/9 = 11.1%), D (3/13 = 23.1%) and E (4/9 = 44.4%) were found to have a lower percentage of samples with detectable SP1 RNA variants than B (9/14 = 64.3%) and C (3/6 = 50%) (Fig. 5B). When all samples regardless of viral load were included, the percentages of detectable spliced RNA positive samples in B (85.7%) and C (80%) were relatively higher than those in A (52.9%), D (41.1%), or E (68.8%) (Fig. 5C). The average percentage of spliced/X gene RNA for the 69 spliced RNA positive samples found in this 100-sample panel was 3.353% (Fig. 5D), approximately 6 times higher than that of spliced/X gene DNA for the 77 spliced DNA positive samples (Fig. 4D). Among the five genotypes, B (7.762 and 8.71%) and C (8.318, 8.511, and 21.878%) had samples carrying higher percentages of SP1 RNA than A (6.026 and 6.607%), D (3.715%) and E (5.888 and 6.166%) (Table S4).

Taken together, compared to A, D or E, genotypes B and C not only generated more detectable SP1 DNA and RNA variants at low replication (≤ 5 log copies/ml), but also produced individual samples having higher percentages of spliced variants during both low and high replications.

## Discussion

A quantitative real-time PCR assay for screening DNA and RNA of HBV SP1 variants was developed for use on the high-throughput, automated Abbott *m*2000 platform. This robust assay detected all nine HBV genotypes and three target regions equally well, and displayed a high sensitivity with an LOD of 80 copies/ml. In fact, 64.6 copies/ml for X gene DNA, 23 copies/ml for SP1 spliced DNA, 30 copies/ml for X gene RNA, and 35.5 copies/ml for SP1 spliced RNA were detected in this study (Tables S2, S3 and S4). By design, the set of primers/probe for the detection of SP1 1.2 kb excision, should not only be able to detect the singly spliced 2.2 kb SP1 variants from the HBV patient plasma samples, but should also theoretically be capable of detecting the doubly spliced variants SP2, SP4 and SP15, with the same 1.2 kb excision. Since the singly spliced SP1 variant was found to be the abundant species that accounted for 50—60% of all splice variants detected in HBV plasma samples^2^, it is assumed that most if not all HBV splice variants detected in this study were SP1 species.

Several studies^31–35^ compared the HBV DNA quantification results determined by the Versant branched DNA (bDNA) assay with those by real-time PCR assays using the S or X gene primers. The viral loads using the S gene primers were overall lower than those by the bDNA assay. In contrast, the assays utilizing the X gene primers correlated well with the bDNA assay. The bDNA assay uses hybridization with multiple regions of the genome and presumably detects both spliced and unspliced HBV DNA (total). These comparative studies suggested that the S primers detected only unspliced DNA, while the X primers, like the bDNA assay, quantified both spliced and unspliced total HBV DNA. Therefore, the X gene was used in the present study to determine the total HBV DNA.

Analysis of the first set of 84 diverse plasma samples for the presence of both SP1 DNA and RNA (Table S2) showed that the level of unspliced DNA or RNA was correlated well with the total concentration of HBV DNA or RNA. This data suggests that unspliced HBV DNA or RNA was the predominant species in circulation, while spliced variants represented a relatively minor population in the plasma of these 84 blood donors whose HCC status was not known. By comparison, the titers of spliced RNA (Fig. 2D) were generally about 1 log copies/ml higher than those of spliced DNA (Fig. 2B). Conversely, the titers of X gene RNA were about 1 log lower than those of X gene DNA (Table S2), which was comparable to previous findings of ~ 1.5 log difference with the HBV pgRNA assay^36,37^. As a result, the average percentage of spliced/X gene RNA was about 5–6 times higher than that of spliced/X gene DNA, making spliced RNA potentially a better biomarker than spliced DNA for monitoring HBV splicing. The relative abundance of SP1 RNA vs SP1 DNA and the fact that RNA production is not impacted by nucleos(t)ide analog (NA) therapy make SP1 RNA a more useful marker than SP1 DNA.

The results presented in this study also reveal that SP1 spliced viral DNA or RNA variants were detectable in most if not all plasma samples with a viral load of > 5 log copies/ml for all five genotypes (A–E) (Figs. 2B,D, 4A and 5A), and higher viral load resulted in increased abundance of splicing, suggesting that during high replication (> 5 log copies/ml) HBV splicing is a frequent event in HBV infection irrespective of genotype. Additionally, it was observed that HBV viral strains in all five genotypes appeared to have similar levels of splicing capacity at high viral loads of > 5 log copies/ml (Figs. 4A and 5A). In contrast, at low viral loads of ≤ 5 log copies/ml the level of HBV splicing varied across the genotypes. The number of samples with detectable SP1 DNA variants was in order of genotypes B (7/10 = 70%) > C (2/6 = 33.3%) > E (2/19 = 10.5%) > D (1/25 = 4%) > A (0/19 = 0%) (Fig. 4B); similarly, the number of detectable spliced RNA positive samples was also in the order of B (9/14 = 64.3%) > C (3/6 = 50%) > E (4/9 = 44.4%) > D (3/13 = 23.1%) > A (1/9 = 11.1%) (Fig. 5B), suggesting that genotypes B and C have the capability to produce more detectable spliced positive samples than A, D or E during low replication (≤ 5 log copies/ml). As for the average spliced/X gene ratios, they were similar across the five genotypes. But at the DNA level, genotype B had individual samples carrying the highest percentages of splice variants (3.02 and 7.079%) (Fig. 4D), and at the RNA level, the order was C (8.318, 8.511 and 21.878%) > B (7.762 and 8.71%) > A (6.026 and 6.607%) > E (5.888 and 6.166%) > D (3.715%) (Fig. 5D), providing evidence that genotypes B and C not only have a greater abundance of spliced positive samples at low replication (≤ 5 log copies/ml), but also generate individual samples having higher percentages of spliced DNA and RNA variants than A, D and E during both low and high replications.

Thus far, our data suggest that the levels of viral replication influence the HBV splicing: during low replication (≤ 5 log copies/ml), the percentage of samples with detectable SP1 DNA or RNA variants is in the order of B > C > E > D > A, while at high replication (> 5 log copies/ml), the levels of SP1 splice variants generated from all five genotypes are similar. The regulatory mechanism by which different HBV genotypes generate different splicing patterns and levels is still unclear. It has been reported that different HBV genotypes exhibit distinct preferences in the usage of splice donor/acceptor sites and thus generate different splicing patterns and levels^28^. Based on the results in this study, it is conceivable that genotypes B and C appear to have more efficient splicing mechanism than A, D or E even when viral replication is low (low viral loads) generating more detectable SP1 spliced variants. But in samples with high viral loads, all genotypes interestingly seem to have similar levels of splicing capacity. Previous studies^14–16^ showed that singly spliced HBV SP1 RNA generated the expression of HBSP, which was linked to an increased risk of the development of HCC by promoting viral replication, protein production and liver fibrosis. Speculatively, because of the constant production of SP1 variants and thus HBSP regardless of the levels of replication, genotypes B and C will likely cause more severe cellular damage than A, D or E, thus resulting in increased risk of HCC development consistent with previous reports that B and C were associated with more severe disease^23,24^ and were more oncogenic^20,25–28^. It has been demonstrated^10^ that the spHBV/wtHBV ratio was significantly elevated in serum samples obtained from patients with severe liver fibrosis or necrosis, thus suggesting a direct link between the spHBV/wtHBV ratio and the severity of liver disease. This observation might be used to explain why B and C which are associated with more severe liver disease were found in this study to generate samples having higher percentages of splice variants than A, D or E. The present study highlights the possible association of constant generation of splice variants regardless of replication level with the higher pathogenicity of genotypes B and C. However, this association must be further investigated in a longitudinal study to evaluate the levels of splice variants during the progression of liver disease.

In summary, we have developed an automated sensitive assay to quantify HBV SP1 DNA and RNA variants in plasma specimens. Most importantly, the assay is consistent for all nine known HBV genotypes and all three target regions. The assay will help expand our understanding of the natural history of HBV infection and the development of HCC, and could be a useful tool in the management of patients with CHB to stratify patients at increased risk for HCC who should receive more intensive screening.

## Materials and methods

### HBV DNA positive plasma samples

Specimens collected from blood donors in Cameroon, Spain, and Thailand were de-identified and obtained according to local regulations in each country at the time of collection between 1997 and 2017, including local IRB approval when required. The study protocols were performed in accordance with the relevant guidelines. This study was approved by the Cameroon National Ethical Review Board, the Faulty of Medicine and Biomedical Science IRB, and the Ministry of Health of Cameroon. Spanish specimens were selected from the Biobank of the Catalonia Blood Bank. An IRB approval was obtained from the Clinical Research Ethics Committee at the Hospital Universitari Vall d’Hebron from Barcelona. An IRB approval was also obtained from the Ethical Review Committee, Thailand Ministry of Public Health. In accordance with study protocols, written informed consents were obtained from human participants (donors) and plasma specimens were collected. Specimens were identified as HBV positive by diagnostic tests done in source countries before performing Abbott RealTi*me* HBV to determine their viral loads and sequence analysis for genotyping through the Abbott Global Surveillance Program^38^. All other plasma specimens sourced from France, Germany, Italy, Spain and Vietnam with known viral loads and genotypes were purchased from Discovery Life Sciences (Huntsville, AL).

### HBV DNA real-time PCR assay

HBV DNA was extracted from 0.5 ml of plasma on the Abbott *m*2000*sp* instrument using the HBV DNA protocol (Abbott Molecular, Des Plaines, IL) and eluted in 50 µl. Three sets of real-time PCR primers and probes were designed to target the conserved regions within the 3’ end of the core gene and the 5’ splice site (the reverse primer RPwt crossing the 5’ splice site), the 3’ end of the core gene and the SP1 splice junction (the reverse primer RPsp crossing the SP1 splice junction), and the 5’ end of the X gene, respectively (Fig. 1B,C). Real-time PCRs were performed by using the TaqMan Universal PCR master mix II (Applied Biosystems, Foster City, CA). Purified HBV DNA (10 µl) was added to a 40 µl PCR mixture containing 25 µl of TaqMan Universal PCR master mix II, 0.3 µM of each primer, and 0.3 µM of fluorogenic probe (the FAM probe for the core gene and the CY5 probe for the X gene). The reaction consisted of one initiating step of 10 min at 95 C, followed by 40 cycles of amplification and reading including 15 s at 95 C, 30 s at 62 C and 90 s at 56 C. The real-time PCR reactions were performed on the Abbott *m*2000*rt* instrument. Data acquisition and analyses were done with Abbott MultiAnalyze v5.04 software. The standard curve for quantification was calculated using serial tenfold dilutions (10^1^–10^6^ copies per PCR reaction) of wtHBV or spHBV plasmids.

### HBV RNA real-time PCR assay

HBV RNA assay was similar to HBV DNA assay with an exception that HBV RNA was extracted from 0.5 ml of plasma on the Abbott *m*2000*sp* instrument using the HBV RNA protocol (Abbott Molecular, Des Plaines, IL) and eluted in 50 µl. Real-time PCRs were performed by using the AgPath-ID One-Step RT-PCR kit (Applied Biosystems, Foster City, CA). Purified HBV RNA (10 µl) was added to a 40 µl PCR mixture containing 25 µl of 2 × RT-PCR buffer, 0.3 µM of each primer, 0.3 µM of fluorogenic probe, and 2 µl of 25 × RT-PCR enzyme mix. The reaction consisted of one initiating step of 30 min at 50 C, followed by 10 min at 95 C and then 40 cycles of amplification and reading including 15 s at 95 C, 30 s at 62 C and 90 s at 56 C.

### Calculation of percentage

A percentage of spliced DNA/X gene total DNA or spliced RNA/X gene total RNA was calculated by dividing the copy number of spliced DNA or RNA by the copy number of X gene total DNA or RNA, and then multiplying the result by 100. The formula used to calculate the percentage was: (the copy number of spliced DNA or RNA/the copy number of total DNA or RNA) × 100%. For example, for sample 846-75 (Table S2) the spliced DNA was 3.37 log copies/ml or 2344.23 copies/ml, and the X gene total DNA was 6.61 log copies/ml or 4073802.8 copies/ml. The percentage of spliced DNA/X gene total DNA for sample 846-75 was (2344.23/4073802.8) × 100% = 0.058%.

### Statistical analysis

Statistical analyses were performed by Student’s T-Tests in Microsoft Excel for comparison between two groups. A value of *p* < 0.05 was considered statistically significant.

## Supplementary Information

Supplementary Information.

## Abbreviations

HBV: Hepatitis B virus
HCC: Hepatocellular carcinoma
pgRNA: Pregenomic RNA
Pol: Polymerase
HBeAg: Hepatitis B e antigen
HBs: Hepatitis B surface protein
wtHBV: Unspliced wild-type HBV
spHBV: Spliced HBV
CHB: Chronic hepatitis B
dHBV: Defective HBV
HBSP: Hepatitis B spliced protein
ORF: Open reading frame
PCR: Polymerase chain reaction
LOD: Limit of detection
