# Supplementary material for: More DNA and RNA of HBV SP1 splice variants are detected in genotypes B and C at low viral replication
Source: Sci Rep. 2021 Dec 13;11:23838. doi: 10.1038/s41598-021-03304-w (PMC8668879; doi:10.1038/s41598-021-03304-w)
Supplement: Supplementary file 1 — Supplementary Information. [file 41598_2021_3304_MOESM1_ESM.pdf]

More DNA and RNA of HBV SP1 splice variants are detected in genotypes B and C at low viral replication

Ka-Cheung Luk, Jeffrey Gersch, Barbara J. Harris, Vera Holzmayer, Dora Mbanya, Silvia Sauleda, Mary A. Rodgers & Gavin Cloherty

Figure S1. Real-time PCR on 18 plasmid constructs (genotypes A – I) carrying either the unspliced (wild-type) or SP1 spliced HBV core gene and 5' end of the X gene. Each plasmid DNA was tested on 10-fold serial dilutions from 10^1 to 10^6 copies/PCR reaction by one of the three primers/probe sets (unspliced/wild-type, spliced/SP1 or X gene). GenBank accession numbers for the 9 HBV genotypes are A-AY161140, B-M54923, C-X75656, D-Y07587, E-X75657, F-AB036910, G-AB375165, H-AP007261, and I-FJ023664.

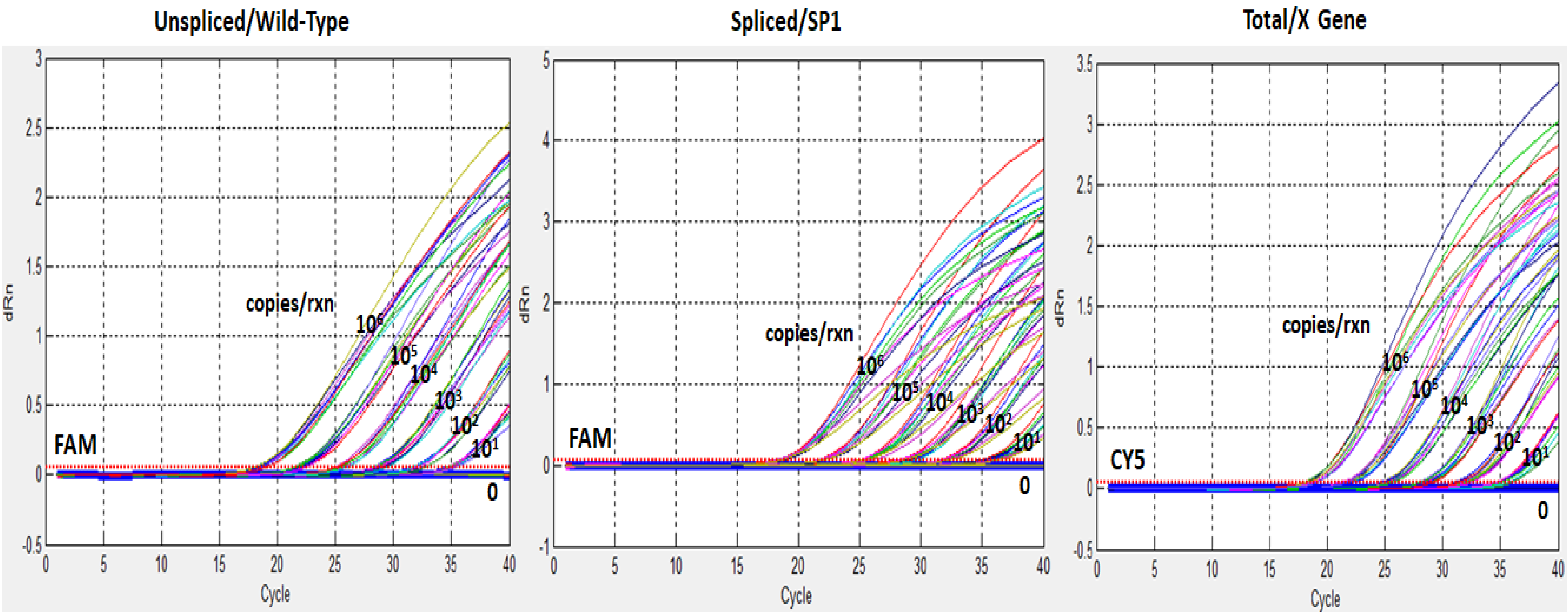

Figure S2. Standard curves from 10-fold serial dilutions (10^1 – 10^6 copies/reaction) of plasmids carrying the X gene, unspliced/wild-type core, or SP1 spliced core sequences of HBV genotypes A (GT-A) or C (GT-C).

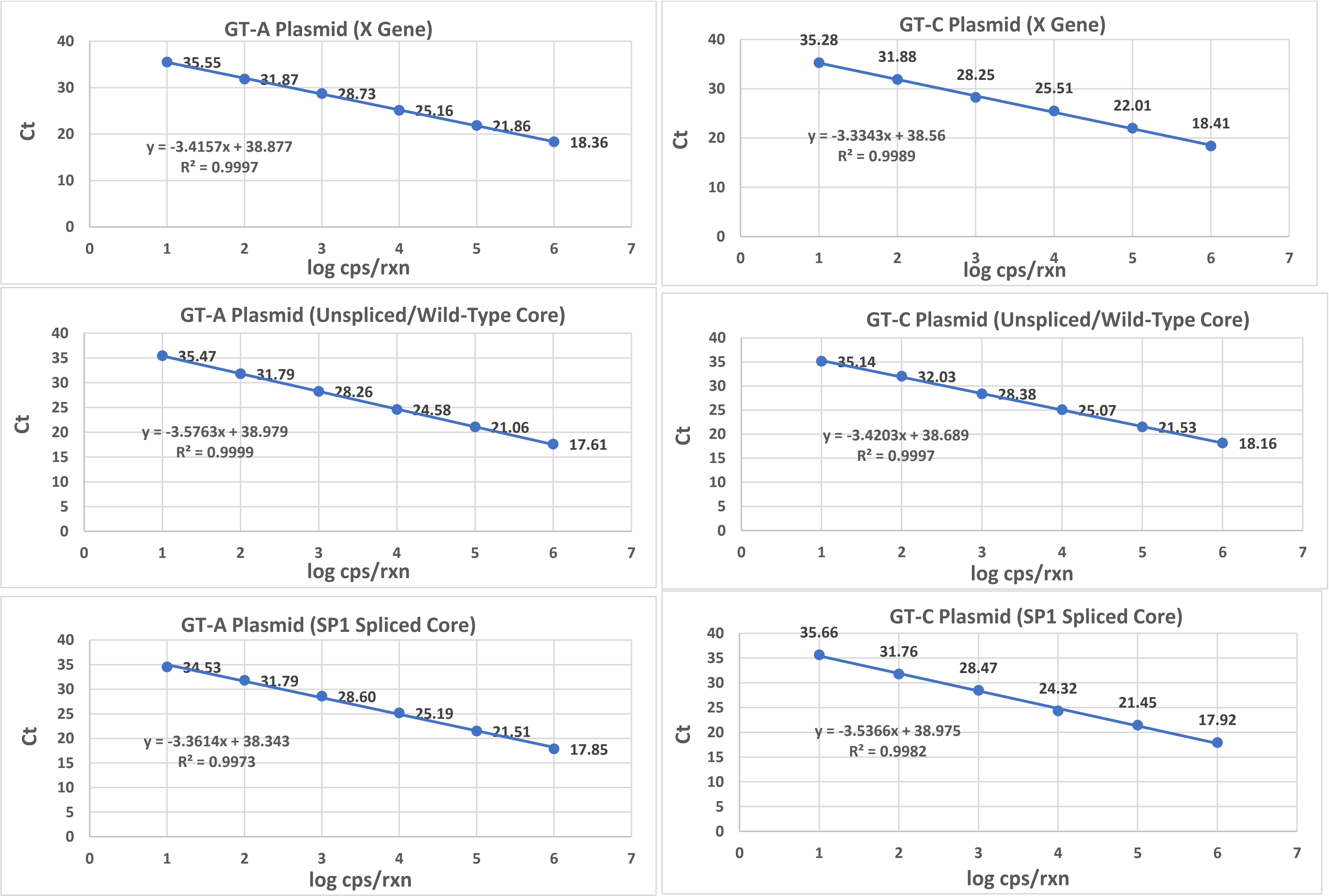

Figure S3. The specificity of primers/probe sets for the detection of plasmid DNA carrying either the unspliced (wild-type) or SP1 spliced core insert.

(A)

Three sets of 10-fold serial dilutions (10^1 – 10^6 copies/rxn) of plasmid BMCX carrying the unspliced (wild-type) core insert were quantified in the presence of 0, 10^2, or 10^4 copies/rxn of spike-in plasmid BMJX carrying the SP1 spliced core insert.

Conclusions: The presence of spike-in plasmid BMJX did not affect the quantitation of plasmid BMCX, proving that the reverse primer RPwt for detecting the unspliced core is specific.

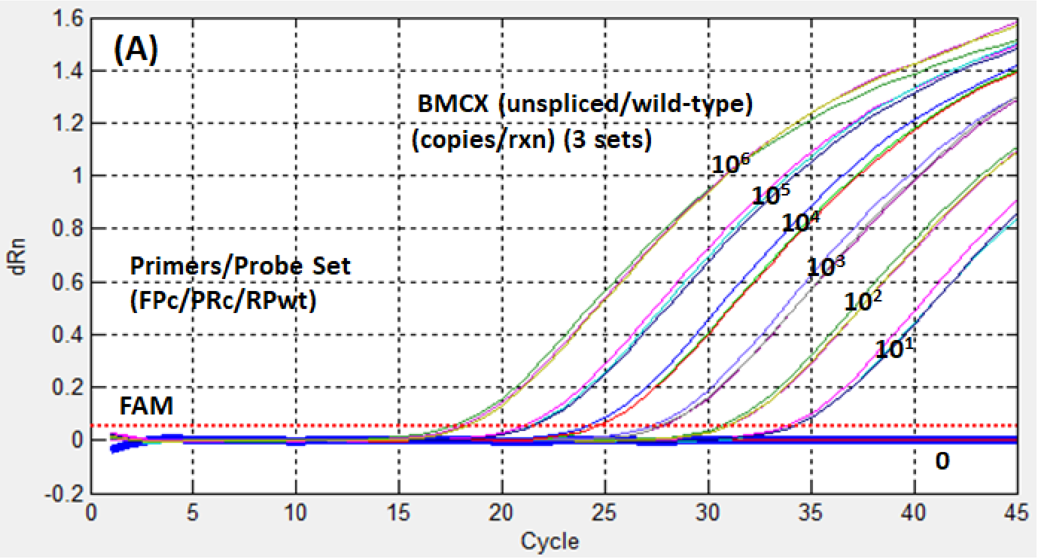

(B)

On the other hand, 10^2 or 10^4 copies/rxn of the spike-in plasmid BMJX in the two reaction sets (10^1 – 10^6 copies/rxn) of plasmid BMCX were detected equally well (7 curves for each titer) when the reverse primer RPsp specific for detecting the spliced core was used.

Conclusions: The presence of BMCX in the reactions, even at 10^6 copies/rxn, did not affect the quantitation of BMJX, proving that the reverse primer RPsp is specific.

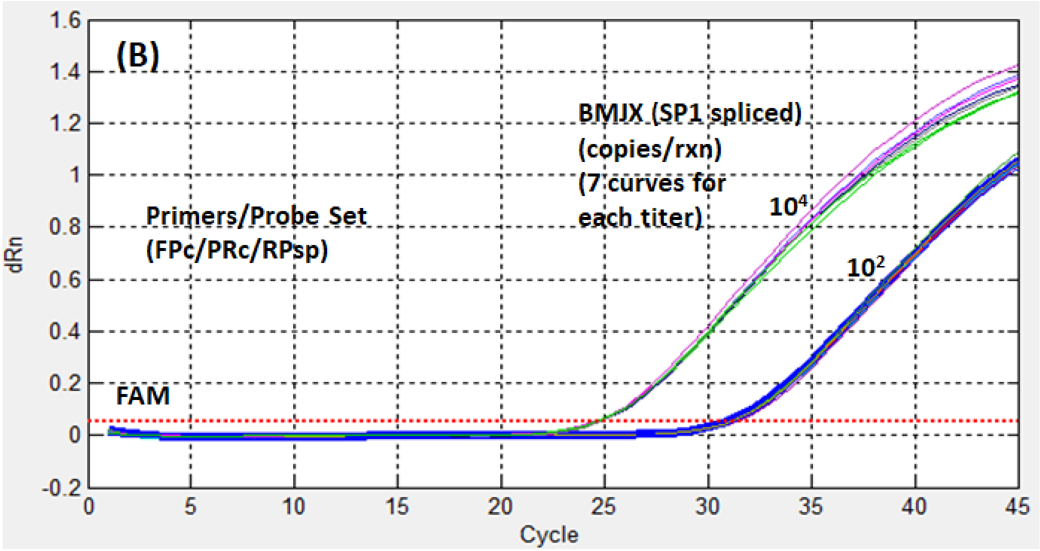

Table S1. Limit of detection and reproducibility on the detection of the X gene DNA of HBV genotype A plasma specimen CHU2247 at low copy dilutions.

Copies/ml	# tested	# detected	% detected	Median Ct	SD	CV (%)
160	24	24	100	34.71	0.42	1.21
80	24	24	100	36.38	0.71	1.95
40	24	20	83.33	37.13	1.79	4.82

Table S2. Screen of 84 HBV DNA positive plasma specimens for the presence of HBV X gene total DNA, unspliced (wild-type) DNA, SP1 spliced DNA, X gene total RNA, unspliced RNA, and SP1 spliced RNA. The calculated percentages of spliced/X gene are also shown. ND: Not detected.

No	Sample ID	Genotype	X Gene DNA log copies/ml	Unspliced DNA log copies/ml	Spliced DNA log copies/ml	Spliced/X Gene DNA (%)	X Gene RNA log copies/ml	Unspliced RNA log copies/ml	Spliced RNA log copies/ml	Spliced/X Gene RNA (%)
1	846-75	A	6.61	6.73	3.37	0.058	5.67	5.91	4.03	2.291
2	827-26	A	6.88	7.14	4.09	0.162	6.11	6.44	4.16	1.122
3	1084-46	A	7.42	7.62	5.19	0.589	6.54	6.64	5.17	4.266
4	U140962	A	6.64	6.90	4.20	0.363	5.59	5.90	4.13	3.467
5	U140681	A	4.09	4.23	ND	0	2.47	2.68	ND	0
6	1485-40	A	4.10	4.47	ND	0	2.79	3.05	ND	0
7	512-09	A	4.05	4.34	ND	0	2.48	2.74	ND	0
8	U141011	A	3.69	3.91	ND	0	3.02	3.46	ND	0
9	U160369A	A	3.42	3.78	ND	0	2.59	2.76	ND	0
10	CHU2247	A	8.49	8.79	5.74	0.178	7.37	7.62	5.55	1.514
11	107-25	A	7.57	7.74	5.83	1.820	7.16	7.34	5.98	6.607
12	B3783-33	A	8.40	8.78	6.09	0.490	8.09	8.46	6.35	1.820
13	ADB1561-12	A	7.85	7.98	6.20	2.239	7.32	7.55	6.10	6.026
14	HBV9005	A	6.22	6.23	3.15	0.085	5.04	4.91	3.38	2.188
15	DLS14-06920	B	7.58	7.88	5.71	1.349	8.17	8.45	6.79	4.169
16	DLS14-06923	B	5.13	5.39	ND	0	4.94	5.30	ND	0
17	DLS14-06937	B	2.09	2.55	ND	0	2.87	3.33	ND	0
18	DLS14-06953	B	4.33	4.68	1.69	0.229	4.02	4.46	2.38	2.291
19	DLS14-06956	B	8.14	8.34	5.80	0.457	8.33	8.78	7.07	5.495
20	DLS14-06887	B	5.80	5.75	3.74	0.871	5.60	5.32	4.03	2.692
21	DLS14-06909	B	5.87	6.22	3.85	0.955	5.41	6.03	4.28	7.413
22	DLS14-06915	B	5.74	6.09	2.59	0.071	5.02	5.51	2.48	0.288
23	DLS14-06945	B	3.64	3.62	2.06	2.630	4.36	4.77	2.99	4.266
24	DLS14-06952	B	4.82	4.79	2.85	1.072	7.20	7.55	5.66	2.884
25	DLS14-08010	B	7.32	7.65	3.94	0.042	6.01	6.32	4.60	3.890
26	DLS14-08032	B	5.27	5.39	3.30	1.072	7.05	7.28	5.34	1.950
27	DLS14-08033	B	4.02	4.16	1.83	0.646	5.91	6.24	3.76	0.708
28	DLS14-07960	B	4.86	4.64	3.71	7.079	8.14	8.38	6.85	5.129

29	DLS14-08026	B	3.69	3.86	ND	0	4.96	5.15	2.42	0.288
30	DLS14-08036	B	6.33	6.57	4.78	2.818	6.57	6.96	5.51	8.710
31	DLS14-08039	B	4.28	4.60	ND	0	4.39	4.62	ND	0
32	DLS14-21191	B	4.05	4.16	2.53	3.020	5.67	6.05	3.98	2.042
33	DLS14-21218	B	7.34	7.72	4.29	0.089	6.75	7.25	4.95	1.585
34	DLS14-21238	B	7.49	7.76	4.38	0.078	7.12	6.56	5.76	4.365
35	DLS14-21267	B	7.09	7.37	3.32	0.017	6.47	6.91	4.50	1.072
36	DLS17-038198	C	7.61	7.84	5.63	1.047	7.43	7.71	6.36	8.511
37	DLS17-038199	C	6.83	7.08	3.46	0.043	5.89	6.06	4.23	2.188
38	DLS17-038205	C	8.44	8.66	5.30	0.072	7.58	7.84	6.04	2.884
39	Thai 13	C	7.29	7.49	4.90	0.407	6.88	6.71	5.49	4.074
40	Thai61	C	8.54	8.85	5.86	0.209	7.62	8.04	6.37	5.623
41	Thai86	C	3.39	3.56	ND	0	1.47	1.55	ND	0
42	Thai89	C	4.27	4.21	1.64	0.234	3.72	3.78	2.26	3.467
43	Thai 220	C	8.40	8.72	5.63	0.170	7.40	7.62	6.02	4.169
44	Thai274	C	5.81	6.00	2.75	0.087	5.03	5.23	3.18	1.413
45	HBV9001	C	9.12	9.35	6.59	0.295	8.09	8.35	7.01	8.318
46	HBV9224	C	6.38	6.57	2.13	0.006	4.63	4.85	2.57	0.871
47	HBV0304	C	8.69	8.91	6.26	0.372	7.94	8.07	6.66	5.248
48	HBV0199	C	4.26	4.29	ND	0	2.88	2.90	ND	0
49	DLS14-06919	B/C	6.31	6.75	4.08	0.589	5.87	6.09	4.61	5.495
50	DLS14-06940	B/C	7.95	8.12	6.19	1.738	8.15	8.18	7.11	9.120
51	DLS14-06942	B/C	5.55	5.87	3.12	0.372	5.30	5.72	3.73	2.692
52	DLS14-06881	B/C	6.25	6.63	2.88	0.043	5.34	5.46	3.36	1.047
53	DLS14-06916	B/C	5.31	4.79	2.64	0.214	7.01	6.34	4.82	0.646
54	DLS14-06922	B/C	7.46	7.64	5.12	0.457	7.03	6.37	5.89	7.244
55	DLS14-06929	B/C	6.67	7.09	3.47	0.063	5.57	5.82	3.69	1.318
56	DLS14-06943	B/C	6.39	6.66	3.90	0.324	6.06	6.43	4.95	7.762
57	DLS14-08006	B/C	6.45	6.93	3.98	0.339	5.92	6.12	4.15	1.698
58	DLS14-08015	B/C	5.96	6.34	2.39	0.027	5.59	5.92	3.43	0.692
59	DLS14-21188	B/C	4.62	4.62	ND	0	5.60	5.30	3.33	0.537
60	DLS14-21252	B/C	7.38	7.66	5.25	0.741	7.13	7.51	6.07	8.710
61	DLS14-21258	B/C	4.95	5.28	2.44	0.309	4.81	4.52	3.29	3.020
62	DLS14-06861	B/C	4.14	4.13	ND	0	4.11	4.03	2.27	1.445
63	DLS14-06889	B/C	6.83	6.93	2.54	0.005	5.49	5.59	3.09	0.398

64	DLS13-11777	D	4.76	5.08	ND	0	2.85	2.87	ND	0
65	DLS14-31179	D	6.88	6.58	ND	0	4.39	4.44	2.44	1.122
66	DLS17-038201	D	8.57	8.73	5.90	0.214	7.35	7.72	5.92	3.715
67	DLS17-038202	D	6.21	6.53	2.90	0.049	5.40	6.01	3.49	1.230
68	DLS17-038203	D	6.44	6.73	1.59	0.001	4.93	5.51	2.07	0.138
69	DLS17-038209	D	7.14	7.31	3.52	0.024	6.36	6.44	4.37	1.023
70	DLS17-038215	D	7.13	7.39	5.07	0.871	6.49	6.59	4.59	1.259
71	DLS17-038212	E	7.07	7.41	3.84	0.059	6.16	6.36	4.71	3.548
72	DLS17-038213	E	5.64	5.66	2.21	0.037	4.61	4.44	2.66	1.122
73	CHU2280	E	5.86	5.99	3.48	0.417	5.05	5.22	3.29	1.738
74	980-1	E	8.74	9.04	5.43	0.049	7.42	7.84	5.70	1.905
75	496-45	E	8.22	8.56	4.20	0.010	6.85	7.04	5.09	1.738
76	1071-42	E	4.58	4.73	2.43	0.708	3.67	3.84	2.22	3.548
77	229-22	E	4.24	4.35	ND	0	2.77	2.94	ND	0
78	230-23	E	3.46	3.60	ND	0	1.56	1.61	ND	0
79	277-11	E	3.92	4.03	ND	0	2.97	3.10	ND	0
80	891-30	E	3.63	4.06	ND	0	2.99	3.24	ND	0
81	U151121A	E	6.87	7.00	4.47	0.398	5.78	5.95	4.41	4.266
82	U160916A	E	7.15	7.38	4.19	0.110	5.80	6.09	4.28	3.020
83	U161114A	E	6.69	6.79	3.98	0.195	5.40	5.55	4.19	6.166
84	U160916	E	7.08	7.20	4.17	0.123	5.51	5.72	4.16	4.467

Table S3. Screen of 147 HBV DNA positive plasma specimens for the presence of HBV X gene total DNA and SP1 spliced DNA. The calculated percentages of spliced/X gene are also shown. Data of 69 samples from Table S2 are in bold, and data of the 78 additional samples are in italics. ND: Not detected.

Genotype	X Gene DNA log copies/ml	Spliced DNA log copies/ml	Spliced/X Gene DNA (%)
**A**	**6.61**	**3.37**	**0.058**
**A**	**6.88**	**4.09**	**0.162**
**A**	**7.42**	**5.19**	**0.589**
**A**	**6.64**	**4.20**	**0.363**
**A**	**4.09**	**ND**	**0**
**A**	**4.10**	**ND**	**0**
**A**	**4.05**	**ND**	**0**
**A**	**3.69**	**ND**	**0**
**A**	**3.42**	**ND**	**0**
**A**	**8.49**	**5.74**	**0.178**
**A**	**7.57**	**5.83**	**1.820**
**A**	**8.40**	**6.09**	**0.490**
**A**	**7.85**	**6.20**	**2.239**
**A**	**6.22**	**3.15**	**0.085**
A	*3.23*	*ND*	*0*
A	*2.45*	*ND*	*0*
A	*3.69*	*ND*	*0*
A	*4.31*	*ND*	*0*
A	*2.97*	*ND*	*0*
A	*3.13*	*ND*	*0*
A	*3.15*	*ND*	*0*
A	*3.98*	*ND*	*0*
A	*3.11*	*ND*	*0*
A	*3.33*	*ND*	*0*
A	*3.30*	*ND*	*0*
A	*4.22*	*ND*	*0*
A	*1.81*	*ND*	*0*
A	*3.75*	*ND*	*0*

A	*6.61*	*3.37*	*0.058*
**B**	**7.58**	**5.71**	**1.349**
**B**	**5.13**	**ND**	**0**
**B**	**2.09**	**ND**	**0**
**B**	**4.33**	**1.69**	**0.229**
**B**	**8.14**	**5.80**	**0.457**
**B**	**5.80**	**3.74**	**0.871**
**B**	**5.87**	**3.85**	**0.955**
**B**	**5.74**	**2.59**	**0.071**
**B**	**3.64**	**2.06**	**2.630**
**B**	**4.82**	**2.85**	**1.072**
**B**	**7.32**	**3.94**	**0.042**
**B**	**5.27**	**3.30**	**1.072**
**B**	**4.02**	**1.83**	**0.646**
**B**	**4.86**	**3.71**	**7.079**
**B**	**3.69**	**ND**	**0**
**B**	**6.33**	**4.78**	**2.818**
**B**	**4.28**	**ND**	**0**
**B**	**4.05**	**2.53**	**3.020**
**B**	**7.34**	**4.29**	**0.089**
**B**	**7.49**	**4.38**	**0.078**
**B**	**7.09**	**3.32**	**0.017**
B	*5.19*	*1.67*	*0.030*
B	*9.40*	*6.97*	*0.372*
B	*4.18*	*2.04*	*0.724*
B	*8.46*	*6.25*	*0.617*
**C**	**7.61**	**5.63**	**1.047**
**C**	**6.83**	**3.46**	**0.043**
**C**	**8.44**	**5.30**	**0.072**
**C**	**7.29**	**4.90**	**0.407**
**C**	**8.54**	**5.86**	**0.209**
**C**	**3.39**	**ND**	**0**
**C**	**4.27**	**1.64**	**0.234**
**C**	**8.40**	**5.63**	**0.170**
**C**	**5.81**	**2.75**	**0.087**

**C**	**9.12**	**6.59**	**0.295**
**C**	**6.38**	**2.13**	**0.006**
**C**	**8.69**	**6.26**	**0.372**
**C**	**4.26**	**ND**	**0**
C	*4.19*	*ND*	*0*
C	*4.47*	*1.74*	*0.186*
C	*6.93*	*3.36*	*0.027*
C	*8.31*	*5.68*	*0.234*
C	*5.33*	*2.04*	*0.051*
C	*8.06*	*5.71*	*0.447*
C	*7.95*	*5.44*	*0.309*
C	*8.78*	*6.21*	*0.269*
C	*8.53*	*5.44*	*0.081*
C	*7.16*	*3.62*	*0.029*
C	*9.24*	*6.02*	*0.060*
C	*7.36*	*4.96*	*0.398*
C	*4.04*	*ND*	*0*
C	*7.82*	*4.91*	*0.123*
C	*8.35*	*5.63*	*0.191*
**D**	**4.76**	**ND**	**0**
**D**	**6.88**	**ND**	**0**
**D**	**8.57**	**5.90**	**0.214**
**D**	**6.21**	**2.90**	**0.049**
**D**	**6.44**	**1.59**	**0.001**
**D**	**7.14**	**3.52**	**0.024**
**D**	**7.13**	**5.07**	**0.871**
D	*5.22*	*2.25*	*0.107*
D	*4.31*	*ND*	*0*
D	*3.24*	*ND*	*0*
D	*5.80*	*2.80*	*0.100*
D	*3.68*	*ND*	*0*
D	*3.82*	*ND*	*0*
D	*4.28*	*ND*	*0*
D	*3.83*	*ND*	*0*
D	*3.32*	*ND*	*0*

D	*4.54*	*ND*	*0*
D	*4.20*	*ND*	*0*
D	*4.13*	*ND*	*0*
D	*3.28*	*ND*	*0*
D	*3.20*	*ND*	*0*
D	*2.71*	*ND*	*0*
D	*3.71*	*ND*	*0*
D	*3.70*	*ND*	*0*
D	*3.86*	*ND*	*0*
D	*3.54*	*ND*	*0*
D	*3.80*	*ND*	*0*
D	*3.65*	*ND*	*0*
D	*3.49*	*ND*	*0*
D	*3.53*	*ND*	*0*
D	*4.27*	*ND*	*0*
D	*4.06*	*1.36*	*0.200*
D	*3.48*	*ND*	*0*
**E**	**7.07**	**3.84**	**0.059**
**E**	**5.64**	**2.21**	**0.037**
**E**	**5.86**	**3.48**	**0.417**
**E**	**8.74**	**5.43**	**0.049**
**E**	**8.22**	**4.20**	**0.010**
**E**	**4.58**	**2.43**	**0.708**
**E**	**4.24**	**ND**	**0**
**E**	**3.46**	**ND**	**0**
**E**	**3.92**	**ND**	**0**
**E**	**3.63**	**ND**	**0**
**E**	**6.87**	**4.47**	**0.398**
**E**	**7.15**	**4.19**	**0.110**
**E**	**6.69**	**3.98**	**0.195**
**E**	**7.08**	**4.17**	**0.123**
E	*3.99*	*ND*	*0*
E	*4.68*	*ND*	*0*
E	*4.25*	*ND*	*0*
E	*6.15*	*ND*	*0*

E	*4.68*	*ND*	*0*
E	*3.35*	*ND*	*0*
E	*7.06*	*4.31*	*0.178*
E	*4.21*	*ND*	*0*
E	*3.46*	*ND*	*0*
E	*3.50*	*ND*	*0*
E	*6.81*	*3.63*	*0.066*
E	*4.18*	*ND*	*0*
E	*5.75*	*3.41*	*0.457*
E	*3.67*	*1.66*	*0.977*
E	*3.33*	*ND*	*0*
E	*3.39*	*ND*	*0*
E	*3.93*	*ND*	*0*
E	*3.95*	*ND*	*0*

Table S4. Screen of 100 HBV DNA positive plasma specimens for the presence of HBV X gene total RNA and SP1 spliced RNA. The calculated percentages of spliced/X gene are also shown. Data of 69 samples from Table S2 are in bold, and data of the 31 additional samples are in italics. ND: Not detected

Genotype	X Gene RNA log copies/ml	Spliced RNA log copies/ml	Spliced/X Gene RNA (%)
**A**	**5.67**	**4.03**	**2.291**
**A**	**6.11**	**4.16**	**1.122**
**A**	**6.54**	**5.17**	**4.266**
**A**	**5.59**	**4.13**	**3.467**
**A**	**2.47**	**ND**	**0**
**A**	**2.79**	**ND**	**0**
**A**	**2.48**	**ND**	**0**
**A**	**3.02**	**ND**	**0**
**A**	**2.59**	**ND**	**0**
**A**	**7.37**	**5.55**	**1.514**
**A**	**7.16**	**5.98**	**6.607**
**A**	**8.09**	**6.35**	**1.820**
**A**	**7.32**	**6.10**	**6.026**
**A**	**5.04**	**3.38**	**2.188**
A	*1.65*	*ND*	*0*
A	*3.08*	*ND*	*0*
A	*3.24*	*ND*	*0*
**B**	**8.17**	**6.79**	**4.169**
**B**	**4.94**	**ND**	**0**
**B**	**2.87**	**ND**	**0**
**B**	**4.02**	**2.38**	**2.291**
**B**	**8.33**	**7.07**	**5.495**
**B**	**5.60**	**4.03**	**2.692**
**B**	**5.41**	**4.28**	**7.413**
**B**	**5.02**	**2.48**	**0.288**
**B**	**4.36**	**2.99**	**4.266**
**B**	**7.20**	**5.66**	**2.884**
**B**	**6.01**	**4.60**	**3.890**
**B**	**7.05**	**5.34**	**1.950**
**B**	**5.91**	**3.76**	**0.708**
**B**	**8.14**	**6.85**	**5.129**

**B**	**4.96**	**2.42**	**0.288**
**B**	**6.57**	**5.51**	**8.710**
**B**	**4.39**	**ND**	**0**
**B**	**5.67**	**3.98**	**2.042**
**B**	**6.75**	**4.95**	**1.585**
**B**	**7.12**	**5.76**	**4.365**
**B**	**6.47**	**4.50**	**1.072**
B	*3.71*	*ND*	*0*
B	*6.51*	*5.40*	*7.762*
B	*7.92*	*6.47*	*3.548*
B	*4.51*	*2.11*	*0.398*
B	*5.60*	*3.91*	*2.042*
B	*4.30*	*2.75*	*2.818*
B	*4.89*	*2.86*	*0.933*
B	*3.87*	*1.55*	*0.479*
B	*3.75*	*ND*	*0*
B	*4.86*	*3.20*	*2.188*
B	*7.01*	*5.61*	*3.981*
B	*5.76*	*3.80*	*1.096*
B	*7.50*	*5.67*	*1.479*
B	*9.67*	*7.85*	*1.514*
**C**	**7.43**	**6.36**	**8.511**
**C**	**5.89**	**4.23**	**2.188**
**C**	**7.58**	**6.04**	**2.884**
**C**	**6.88**	**5.49**	**4.074**
**C**	**7.62**	**6.37**	**5.623**
**C**	**1.47**	**ND**	**0**
**C**	**3.72**	**2.26**	**3.467**
**C**	**7.40**	**6.02**	**4.169**
**C**	**5.03**	**3.18**	**1.413**
**C**	**8.09**	**7.01**	**8.318**
**C**	**4.63**	**2.57**	**0.871**
**C**	**7.94**	**6.66**	**5.248**
**C**	**2.88**	**ND**	**0**
C	*8.19*	*7.53*	*21.878*

C	*3.55*	*ND*	*0*
**D**	**2.85**	**ND**	**0**
**D**	**4.39**	**2.44**	**1.122**
**D**	**7.35**	**5.92**	**3.715**
**D**	**5.40**	**3.49**	**1.230**
**D**	**4.93**	**2.07**	**0.138**
**D**	**6.36**	**4.37**	**1.023**
**D**	**6.49**	**4.59**	**1.259**
D	*2.84*	*ND*	*0*
D	*2.09*	*ND*	*0*
D	*2.83*	*ND*	*0*
D	*2.35*	*ND*	*0*
D	*2.70*	*ND*	*0*
D	*4.80*	*1.55*	*0.056*
D	*3.02*	*ND*	*0*
D	*3.11*	*ND*	*0*
D	*3.15*	*ND*	*0*
D	*2.75*	*ND*	*0*
**E**	**6.16**	**4.71**	**3.548**
**E**	**4.61**	**2.66**	**1.122**
**E**	**5.05**	**3.29**	**1.738**
**E**	**7.42**	**5.70**	**1.905**
**E**	**6.85**	**5.09**	**1.738**
**E**	**3.67**	**2.22**	**3.548**
**E**	**2.77**	**ND**	**0**
**E**	**1.56**	**ND**	**0**
**E**	**2.97**	**ND**	**0**
**E**	**2.99**	**ND**	**0**
**E**	**5.78**	**4.41**	**4.266**
**E**	**5.80**	**4.28**	**3.020**
**E**	**5.40**	**4.19**	**6.166**
**E**	**5.51**	**4.16**	**4.467**
E	*3.31*	*2.08*	*5.888*
E	*1.79*	*ND*	*0*
